# Clinical management of pheochromocytoma and paraganglioma in Singapore: missed opportunities for genetic testing

**DOI:** 10.1002/mgg3.313

**Published:** 2017-07-20

**Authors:** Winston Hong Wern Chew, Eliza Courtney, Kok Hing Lim, Shao Tzu Li, Yanni Chen, Min Han Tan, Alexander Chung, Joan Khoo, Amos Loh, Shui Yen Soh, Prasad Iyer, Lih Ming Loh, Joanne Ngeow

**Affiliations:** ^1^ Cancer Genetics Service Division of Medical Oncology National Cancer Centre Singapore Singapore City 169610 Singapore; ^2^ Department of Pathology Singapore General Hospital Singapore City 169610 Singapore; ^3^ Division of Surgery Singapore General Hospital Singapore City 169610 Singapore; ^4^ Department of Endocrinology Changi General Hospital Singapore City 529889 Singapore; ^5^ Department of Paediatrics Surgery KK Women's and Children's Hospital Singapore City 229899 Singapore; ^6^ Division of Paediatrics KK Women's and Children's Hospital Singapore City 229899 Singapore; ^7^ Department of Paediatric Subspecialties Haematology‐Oncology Service KK Women's and Children's Hospital Singapore City 229899 Singapore; ^8^ Department of Endocrinology Singapore General Hospital Singapore City 169610 Singapore

**Keywords:** Genetic counselling, genetic testing, paraganglioma, pheochromocytoma

## Abstract

**Background:**

Pheochromocytomas and paragangliomas (PPGLs) are neuroendocrine tumors of the adrenal glands and paraganglia, occurring sporadically or as a range of hereditary tumor syndromes. About 30% of PPGLs are attributed to germline mutations. Clinical presentation, including localization, malignant potential, and age of onset, varies depending on the genetic background. Genetic testing for PPGLs is not well studied in Southeast Asia. We reviewed clinical management of PPGLs in Singapore, highlighting current gaps in clinical practice.

**Methods:**

Medical records of patients with PPGLs between 2005 and 2016 were reviewed. Diagnosis was confirmed histologically and stratified into sporadic or familial/syndromic (FS).

**Results:**

Twenty‐seven (21.8%) patients were referred to the Cancer Genetics Service (CGS). FS PPGLs (18.5%) and extra‐adrenal PPGLs (58.1%) incidences were higher than previous studies. Referrals were lower for sporadic PPGLs compared to FS PPGLs (3.7% vs. 100%). Referrals were highest at diagnosis age <20 years old (80%) and decreased with increasing age; ≥20–<40 years old (32.1%), ≥40–<60 years old (10.6%). Genetic testing was taken up in 12/27 (44.4%) patients of which 7/12 (58.3%, *3 SDHB, 2 SDHD, 2 VHL*) had germline mutations.

**Conclusion:**

Opportunities for genetic testing are frequently missed due to low referral rates in patients with apparently sporadic PPGLs, particularly between ages 20‐60.

## Introduction

Pheochromocytomas (PCC) and paragangliomas (PGL), together abbreviated as (PPGLs), are rare neural crest derived tumors forming from the adrenal medulla and extra‐adrenal paraganglia, respectively. Some have a catecholamine secretory nature that could instigate fatal cardiovascular episodes (Dahia 2014); yet, as many as 50% of PCC are unfortunately only found at autopsy (Manger 2006). Additionally, postoperative PPGLs have a 15% relapse risk, with most being metastatic recurrence that can occur decades after surgery, implying a need for effective screening and cheap long‐term follow‐up management (Amar et al. 2012). While various scoring systems have been suggested to predict the malignant potential of PPGLs, the low sensitivity of these tools make their utilization unreliable (Lenders et al. 2014).

With the advent of next‐generation sequencing technologies and PPGLs seeing increased attribution to germline mutations; 30–40% in 2015 versus 10% in 2000 (Favier et al. 2015), genetic testing is quickly becoming a viable adjuvant means of screening and managing PPGLs (Favier et al. 2015). Genetic testing for all cases of PPGLs is part of standard care (Lenders et al. 2014) as it not only provides clinicians more information to guide their patient's management (Amar et al. 2007), but also helps determine the need for early screening in relatives of patients with suspected hereditary PPGLs.

Currently known susceptibility genes include *RET* proto‐oncogene, *VHL*, and *NF1* tumor‐suppressor genes, succinate dehydrogenase complex subunits (*SDHA‐D*), succinate dehydrogenase complex assembly factor (*SDHAF2*)*,* and transmembrane protein 127 gene (*TMEM127*) (Welander et al. 2011), among other. A number of major studies have previously characterized the genotype–phenotype relationship of these genes and reported varying degrees of penetrance between different germline mutations (Neumann et al. 2002; Benn et al. 2006; Ricketts et al. 2010).

Despite the advances in understanding between PPGLs and germline mutations, there is a paucity of studies on genetic testing and management of PPGLs in Southeast‐Asia (Khadilkar et al. 2016). In Singapore, clinical cancer genetics services were not well established prior to 2014. We describe here a “real‐world” experience with PPGL management to understand the referral patterns and uptake of genetic testing in Singapore to better understand gaps in clinical management.

## Methods

### Patients

All data were consented with the approval and institutional oversight of SingHealth Centralised Institutional Review Board. This study retrospectively reviewed 124 consecutive patients with PPGLs that were seen in the largest tertiary hospital in Singapore, Singapore General Hospital, between 2005 and 2016. PPGLs diagnosis was confirmed by histological examination post tumor resection and localized with anatomical imaging or functional imaging; iodine^123^‐labeled metaiodobenzylguanidine scintigraphy (I‐MIBG), fluorodeoxyglucose Pet/CT (FDG), or Ga‐Dotatate Pet/CT. Clinical information gathered included age of diagnosis, sex, tumor location, size, bilaterality, multiplicity, biochemical profile and malignancy. Malignancy was defined as the presence of distant extra‐adrenal/extra‐paraganglia metastasis (Eisenhofer et al. 2004). Patients were stratified into two groups; (1) “Sporadic” patients with tumors that were incidentally found on imaging; (2) “Familial/Syndromic” (FS) patients with symptoms matching the criteria of known syndromes or had a positive family history.

A further review on genetic testing outcomes was done on 27 patients from the study population who were seen at National Cancer Centre Singapore, Cancer Genetic Service (CGS). Genetic analysis used next‐generation whole exome sequencing technologies and known susceptible germline mutations (that included *RET, VHL, NF1, SDHA, SDHB, SDHD, SDHAF2,* and *TMEM127)* were investigated.

### Statistical analysis

Statistical analysis was performed with IBM SPSS Statistical software version 22, where *P* < 0.05 was deemed statistically significant.

## Results

### Patient characteristics

The clinical characteristics of this cohort is depicted in Table 1. Overall there were 58/124 (46.8%) males and 66/124 (53.2%) females with a median diagnosis age of 51.5 years (7–90). Adrenal tumors were seen in 55/124 (44.4%) patients, while 72/124 (58.1%) patients had extra‐adrenal tumors, of which three patients had both adrenal and extra‐adrenal tumors. Sixty‐nine out of eighty‐seven (79.3%) tumors were secretory in nature. Bilateral and multifocal tumors were present in 6/124 (4.8%) and 12/124 (9.7%) patients, respectively. Finally, 23/124 (18.5%) patients were classified as FS, while 101/124 (81.5%) were sporadic.

**Table 1:** Clinical characteristics of patients referred to CGS versus patients who were not referred

Characteristics	All patients		Patients referred to CGS		Patients not referred		*P* value
	*N*	(%)	*N*	(%)	*N*	(%)	
No.	124		27	21.8	97	78.2	
Sex							
Male	58	46.8	7	25.9	51	52.6	**0.006**
Female	66	53.2	22	81.5	44	45.4	
Age at diagnosis (Years)							
Median	51.5		36.7		53		**<0.001**
Range	7–90		7–75		13–90		
Tumor size (cm)							
Median	3.7		3.8		3.5		0.251
Range	0.1–12		1–9		0.1–12		
Adrenal tumors	58	46.8	16	59.2	42	43.3	0.191
Extra‐adrenal tumors	69	55.7	11	40.7	58	60.0	**0.085**
Secreting tumors	69/87	79.3	19/22	86.3	50/65	76.9	0.543
Bilateral tumors	6	4.8	2	7.4	4	4.1	0.610
Multifocal tumors	12	9.7	6	22.2	6	6.2	**0.022**
Malignant tumors	17	13.7	8	29.6	9	9.3	**0.012**
Familial/Syndromic tumors	23	18.5	23	85.2	0	0	**<0.001**
Sporadic tumors	101	81.5	4	14.8	97	100	

Bold P values are statistically significant.

Radiologically, 120/124 (96.8%) patients had anatomical imaging done that included 37/124 (29.8%) patients who had additional functional imaging, of which 6 patients had both I‐MIBG and FDG/Ga‐DOTATATE PET/CT. Of the 17 I‐MIBG scans, 11/17 (64.7%) scans were sensitive in detecting PPGLs. Additionally, 26/37 (70.2%) patients had FDG/Ga‐DOTATATE PET/CT. PPGLs were detected in 25/26 (96.2%) patients, the remaining one PPGL was only detected on CT scans. With regard to metastatic detection, I‐MIBG detected 2/6 (33.3%) metastatic PPGLs, while FDG/Ga‐Dotatate PET/CT detected 11/11 (100%) metastatic PPGLs.

### Patients referred to CGS

Of the 124 patients, 27/124 (21.8%) patients were referred to CGS with 12/27 (44.4%) eventually undergoing genetic testing. As depicted in Table 2, 7/12 (58.3%) patients had positive germline mutations (3 *SDHB*, 2 *SDHD*, 2 *VHL*) and an additional patient was identified to carry a variant of uncertain significance (VUS). Patients were referred from a variety of subspecialties: one each from radiation oncology, general surgery, neurosurgery, and hepato‐pancreato‐biliary surgery; four from pediatrics; five from surgical oncology; and eight each from urology and endocrinology. The median time between first diagnosis of PPGLs and first consultation at CGS was 282 days with an interquartile range of 62–585 days.

**Table 2:** Identified germline mutations and presentation

Gene	cDNA nucleotide	Amino acid change	Exon	Clinical features		
				Age (Years)	Tumor localization	Functionality
*SDHB*	c.136C>T	P.Arg46*	2	17	Metastatic (Retroperitoneal)	Syndromic
	c.136C>T	p.Arg46Ter	2	56	Unifocal (Neck)	Syndromic
	c.620_621delTG	p.Leu207Argfs*14	6	19	Metastatic (Retroperitoneal)	Syndromic
*SDHD*	c.10dupC	p.Leu4Profs*65	1	11	Unifocal (Adrenal)	Syndromic
	c.242delC	p.Pro81Argfs*5	3	37	Multifocal (Bilateral Neck + adrenal)	Syndromic
*VHL*	c.191G>C	p.Arg64Pro	1	35	Unifocal (Adrenal)	Syndromic
	c.499C>T	p.R167W	3	17	Unifocal (Adrenal)	Sporadic
*EGNL1*	c.319G>T (VUS)	p.Ala107Ser	1	60	Unifocal (Bladder)	Sporadic

Table 1 outlines differences between patients referred to CGS against those that were not. Only 8/17 (47%) patients with malignant PPGLs were referred with 4/17 (23.5%) patients undergoing genetic testing. Additionally, there was also a large difference in the proportion of FS versus SPR patients who were referred to CGS, with all FS PPGLs patients (100%) being referred with only 4/107 (3.7%) SPR PPGLs being referred.

Predictors of referral to the CGS included female sex (Median: 81.5% vs. 45.4%, *P* = 0.006), younger age of diagnosis (Median: 36.7 vs. 53 years old, *P* < 0.001), PPGLs that were multifocal (Median: 22.2% vs. 6.2%, *P* = 0.022), malignant (Median: 29.6% vs. 9.3%, *P* = 0.012), or FS (Median: 85.2% vs. 0%, *P* < 0.001).

**Figure 1:**
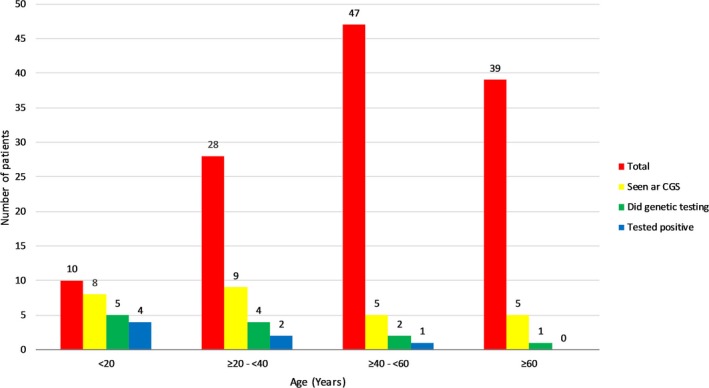
Graph of PGL/PCC by age of diagnosis between overall cohort, patients referred to CGS, patients who undergo genetic testing and patients with tested positive germline mutation.

The proportion of patients per diagnosis age group seen at CGS is highest in the <20 years old category with 80% (8/10) as reflected by Figure 1. This progressively decreased to 32.1% (9/28), 10.6% (5/47), and 12.8% (5/39) patients from diagnosis age groups ≥20–<40, ≥40–<60, and ≥60 years old, respectively. This trend followed for patients who eventually underwent genetic testing; Patients who were diagnosed below the age of 20 had the highest rate of genetic testing at 50% (5/10). This subsequently decreased to 14.3% (4/28), 4.3% (2/47), and 2.6% (1/39) for the group of patients that were diagnosed at age ≥20‐<40, ≥40‐<60 and ≥60 years old respectively.

The germline mutation spectrum seen in patients who had genetic testing and the clinicopathological factors that they presented with is depicted in Table 2. Three *SDHB* missense mutations were found; twice in codon 136 of exon 1 and a frameshift mutation in codon 620 of exon 6. Common characteristics within the group were metastatic PPGLs. Two *SDHD* mutations were also found; these consisted of frameshifts at codon 10 of exon 1 and codon 242 of exon 3, respectively. Two *VHL* mutations were found with both having a missense at codon 191 of exon 1 and codon 499 of exon 3, respectively.

## Discussion

In this first Singaporean study, we provide a picture of the clinical characteristics and diagnostic sensitivity of patients with PPGLs in a South‐East Asian population of both children and adults, thereby highlighting current gaps in management. The incidence of malignant PPGLs and common sites of metastasis (spine, liver, lungs) in our cohort of patients is similar to other studies done previously (Goldstein et al. 1999; Neumann et al. 2002; Mannelli et al. 2009; Khadilkar et al. 2016).

In line with previous studies, F‐FDG/Ga‐DOTATATE Pet/CT scans are the most sensitive at detecting metastatic PPGLs, compared to I‐MIBG which only detected metastatic PPGLs a third of the time (Mojtahedi et al. 2014) However, only three‐quarter of patients with malignant PPGLs and a quarter of patients in the study cohort had any functional imaging done, displaying the lack of uptake of the available imaging modality and variable practice among clinicians in the absence of genetic analysis to guide specific surveillance and postoperative management.

Knowledge regarding the genetic background of patients with PPGLs has the potential to improve management by giving a more accurate risk stratification. While most PPGLs have a low risk of malignancy, patients with *SDHB* mutations have higher risks of malignancy, and conversely patients with RET germline mutations have a much lower risk of malignancy (Amar et al. 2007).

It is important to test both FS and sporadic PPGL patients as family history is not always a useful indicator for positive germline mutation (Benn et al. 2006). This issue lies partly due to phenotypic expressions being complicated by reduced penetrance and parent of origin effects. Another cause includes the difficulty in eliciting an accurate family history due to gaps or errors in the patient's knowledge. This is particularly seen in *SDH* mutations, where family history may be absent (Welander et al. 2011).

Current genetic testing algorithms are based either on the 2014 American College of Medical Genetics (Hampel et al. 2015) or the 2014 Endocrine Society Clinical Practice Guidelines which were formulated by extensive studies by the European Network for the Study of Adrenal Tumours (ENSAT) (Gimenez‐Roqueplo et al. 2006). Both guidelines recommend that all patients with PPGLs should consider genetic testing as part of their management. However, despite similarities in patient population to other studies (Amar et al. 2005; Mannelli et al. 2009), only 20% of patients with PPGL were referred for genetic counselling, with less than half of these eventually agreeing for testing. Barriers for genetic testing in Asia are multifactorial and beyond the scope of this paper, however cost of testing is a major concern for patients (Li et al. 2016).

Based on subgroup analysis of the clinicopathological features of patients, there was a significant group of patients who were not referred for genetic management despite having clinical features that made them likely to be germline mutation carriers of susceptibility genes. These groups included four patients with bilateral adrenal PPGL, six patients with multiple PPGL, nine patients with malignant PPGL, a considerable number of young patients, at 13 years at age of diagnosis and further cases younger than 30, and 58 patients with extra‐adrenal PPGL. In these patients, identifying those that are germline mutation carriers is the cornerstone for adequate management.

Our data suggest that clinicians were correctly recognizing the need to refer young patients (<20 years old) with PPGLs for genetic testing and referring FS PPGLs, but they were less likely to refer older sporadic PGLs in those patients diagnosed over age 20. Neumann et al. reported finding germline mutations for one fifth of those falling in this age group with sporadic PPGLs (Neumann et al. 2002). Based on current guidelines, all patients with PPGLs should consider genetic testing; yet, most do not even have a referral for a consultation. This suggests that there are significant missed opportunities for intervention particularly in older patients. Targeted physician and patient education is currently planned to improve current practice.

## Conclusion

Genetic testing in Singapore for this group of patients is still underutilized, particularly in those diagnosed above age 20 and with apparently sporadic PPGL. This highlights the need for improvements for referral to cancer genetic clinics and identifies an area for targeted intervention, both at the clinician and patient level. For this to be achieved effectively, further research is warranted to explore where the barriers to referral are for this target group including improving patient and physician awareness and reducing cost of genetic testing on medical grounds via subsidies.

## Conflict of Interest

None declared.
